# Genetic Diversity and Population Structure of Races of *Fusarium oxysporum* Causing Cotton Wilt

**DOI:** 10.1534/g3.120.401187

**Published:** 2020-07-20

**Authors:** Hannah C. Halpern, Peng Qi, Robert C. Kemerait, Marin T. Brewer

**Affiliations:** * Department of Plant Pathology, University of Georgia, Athens, Georgia 30602; † Department of Plant Biology, University of Georgia, Athens, Georgia 30602; ‡ Department of Plant Pathology, University of Georgia, Tifton, Georgia 31793

**Keywords:** fungi, races, FOV, genotyping-by-sequencing, population genetics, plant pathology

## Abstract

To better understand the evolution of virulence we are interested in identifying the genetic basis of this trait in pathogenic fungi and in developing tools for the rapid characterization of variation in virulence among populations associated with epidemics. *Fusarium oxysporum* f. sp. *vasinfectum* (FOV) is a haploid fungus that causes devastating outbreaks of Fusarium wilt of cotton wherever it is grown. In the United States, six nominal races and eleven genotypes of FOV have been characterized based on the translation elongation factor (*EF-1α*) gene and intergenic spacer region (IGS), but it is unclear how race or genotype based on these regions relates to population structure or virulence. We used genotyping-by-sequencing to identify SNPs and determine genetic diversity and population structure among 86 diverse FOV isolates. Six individuals of *Fusarium oxysporum* closely related to FOV were genotyped and included in some analyses. Between 193 and 354 SNPs were identified and included in the analyses depending on the pipeline and filtering criteria used. Phylogenetic trees, minimum spanning networks (MSNs), principal components analysis (PCA), and discriminant analysis of principal components (DAPC) demonstrated that races and genotypes of FOV are generally not structured by *EF-1α* genotype, nor are they monophyletic groups with the exception of race 4 isolates, which are distinct. Furthermore, DAPC identified between 11 and 14 genetically distinct clusters of FOV, whereas only eight *EF-1α* genotypes were represented among isolates; suggesting that FOV, especially isolates within the widely distributed and common race 1 genotype, is more genetically diverse than currently recognized.

*Fusarium oxysporum* (*Fo*) is an agriculturally important fungus that causes disease on over 120 plant hosts (Edel-Hermann and Lecomte 2019). *Fo* is subcategorized into *formae speciales* (ff. spp.), with each *forma specialis* (f. sp.) defined by the host on which it can cause disease. Most *formae speciales* of *Fo* are soilborne plant pathogens that cause vascular wilt diseases. Examples include: *Fusarium oxysporum* f. sp. *niveum*, which causes the globally important Fusarium wilt of watermelon (Martyn 2014); and *Fusarium oxysporum* f. sp. *cubense*, which causes the devastating Panama disease of banana (Ploetz 1994). Interestingly, the *formae speciales* of *Fo* are often polyphyletic. Based on sequence similarity for the translation elongation factor (*EF-1α*) gene and the intergenic spacer region (IGS), individuals from different *formae speciales* may be more closely related to each other than individuals in the same *forma specialis*, suggesting that pathogenicity to certain plant hosts has independently arisen multiple times from distinct lineages (O’Donnell *et al.* 2009). This most likely resulted from the horizontal transfer of lineage-specific pathogenicity chromosomes, also called accessory chromosomes (Ma *et al.* 2010). *Fusarium oxysporum* is characterized by the presence of lineage-specific (LS) chromosomes, which are enriched with transposable elements (TE’s) and genes for small secreted proteins. The LS chromosomes are also highly divergent among different *formae speciales*, suggesting that they play a role in host-specialization (Ma *et al.* 2010; de Sain and Rep 2015). *Fo* is haploid, and considered asexual since it reproduces clonally, sexual reproduction has never been observed, and is not known to undergo meiotic recombination (Kang *et al.* 2014).

FOV, the *forma specialis* that causes Fusarium wilt of cotton, has recently re-emerged in the southeastern United States (Atkinson 1892; Whitaker *et al.* 2016). In the southeastern U.S., Fusarium wilt of cotton was historically a relatively minor disease problem because effective disease management practices - especially the management of plant-pathogenic nematodes which aggravate Fusarium wilt symptoms - were used (Jorgenson *et al.* 1978; Garber *et al.* 1979). In the past decade, despite the same disease management practices being followed, Fusarium wilt has become increasingly prevalent in the southeastern United States, with some outbreaks occurring early in the cotton-growing season and causing severe damage to seedlings (Collins *et al.* 2013; Whitaker *et al.* 2016).

FOV has been studied for over a century, but there are still knowledge gaps in the pathogen’s genetic diversity (Atkinson 1892). Eight pathogenic races of FOV were reported between 1958 and 1985, and although these races are widely regarded as invalid (Armstrong and Armstrong 1958; Armstrong and Armstrong 1960; Ibrahim 1966; Armstrong and Armstrong 1978; Chen *et al.* 1985; Davis *et al.* 2006; Bell *et al.* 2017), they are widely used in the cotton industry for characterizing FOV. In plant pathology, a race refers to a pathogen’s ability to cause disease on its host and implies a gene-for-gene interaction between host and pathogen (Anderson *et al.* 2010). A set of differential host cultivars with unique combinations of resistance genes are frequently used to discriminate pathogenic races. The races of FOV, however, were characterized based on isolates’ virulence not only on a differential set of cotton cultivars, but also on many non-host plants including tobacco, soybean, and okra (Armstrong and Armstrong 1960; Armstrong and Armstrong 1978; Chen *et al.* 1985). Additionally, many of the cotton cultivars that were originally used to differentiate races of FOV have not been maintained, and differential virulence reactions are not reproducible on modern cotton cultivars (Davis *et al.* 2006; Holmes *et al.* 2009; Bell *et al.* 2017;). Furthermore, multigene genealogies, pathogenicity tests, and other bioassays demonstrated that races 3 and 5, as well as 4 and 7, are genetically and phenotypically indistinguishable and should therefore be recognized as single groups (Nirenberg *et al.* 1994; Hering *et al.* 1999; Skovgaard *et al.* 2001). As a result, only six races of FOV are recognized today.

Each race of FOV is associated with a unique sequence at the *EF-1α* locus, with the exception of races 1 and 6, but race 6 is apparently limited to South America; *EF-1α* sequence is the primary tool used to characterize races of FOV today (Skovgaard *et al.* 2001; Kim *et al.* 2005; Cianchetta *et al.* 2015). “Genotype” would be a more valid term than “race” to describe these polymorphisms, but the term “race” is still used nominally in the United States cotton industry (Davis *et al.* 2006). In addition to the six nominal races, there are four other genotypes of FOV characterized by unique *EF-1α* sequences, referred to as LA108, LA110, LA112, and LA127; and one genotype, MDS-12, that is identical to FOV race 4 in *EF-1α* sequence but unique in intergenic spacer region (IGS) sequence (Holmes *et al.* 2009; Bennett *et al.* 2013).

Although housekeeping gene sequences, especially *EF-1α*, have been used to characterize FOV isolates in the United States, there is some evidence that these polymorphisms do not fully explain the genetic diversity and evolutionary history of FOV. For example, a phylogeny generated from *EF-1α*, mitochondrial small subunit ribosomal DNA (mtSSU rDNA), nitrate reductase (*NIR*), and phosphate permease-like protein (*PHO*) sequences, showed race 2 to be a polyphyletic group (Skovgaard *et al.* 2001). A separate phylogeny generated from IGS, *PHO*, *EF-1α*, and beta-tubulin (*BT*), showed that MDS-12 is also polyphyletic (Cianchetta *et al.* 2015). Given that FOV is considered a highly diverse pathogen, there may be other polymorphisms being overlooked in characterizations based on housekeeping gene sequences.

Genotyping-by-sequencing (GBS) is a form of reduced-representation genome sequencing in which genomic DNA is digested with a restriction enzyme, and short fragments are amplified and sequenced via next-generation sequencing (Elshire *et al.* 2011; Andrews *et al.* 2016). Depending on coverage, this approach allows for the identification of hundreds to thousands of single nucleotide polymorphisms (SNPs) among a large sample of individuals, thus providing high resolution of genetic differences among individuals. GBS has previously been used in population analyses of plant pathogens to identify novel genetic diversity in high-diversity organisms, identify cryptic sexual recombination among clonal pathogens, and conduct genome-wide-association-studies (GWAS) linking quantitative trait nucleotides (QTNs) with pathogen virulence and mycotoxin production (Milgroom *et al.* 2014; Hansen *et al.* 2016; Talas *et al.* 2016).

The goal of this study was to characterize the genetic diversity, evolutionary history, and population structure of FOV using GBS to identify SNPs among diverse isolates of FOV. Additionally, we wanted to replicate our GBS data processing pipeline using two different reference genomes - one publicly available annotated reference genome, and one reference assembled *de novo* from the raw GBS data - to assess the effect of the reference on population analyses.

## Materials and Methods

### Isolate collection

One-hundred-and-fourteen single- spore cultures of FOV were isolated from symptomatic plants throughout Georgia cotton fields (Table S1), following the protocol described by da Silva *et al.* (2019). Isolates were genotyped for FOV race type based on their translation elongation factor (*EF-1α*) sequence, using the primers EF1 and EF2 (O’Donnell *et al.* 1998; Cianchetta *et al.* 2015). Each PCR reaction consisted of 1.25 μl 2.5 mM each dNTP’s, 1.25 μl 10× ExTaq buffer (Takara Bio USA), 0.56 μl of each 10 μM primer, 0.3 μl ExTaq (Takara Bio USA), and 1 μl of genomic DNA (10 - 300 ng/μl). Amplification was conducted in a thermal cycler (PTC-100; MJ Research, Watertown, MA) under the following conditions: 95° for 1 min; 40 cycles of 95° for 30 sec, 55° for 30 sec, and 72° for 1 min; and a final extension of 72° for 5 min (Cianchetta *et al.* 2015). Amplification of the *EF-1α* locus was confirmed by 1% agarose gel electrophoresis, and PCR products were purified with an ExoSAP-IT kit (Thermo Fisher Scientific, Waltham, MA) per manufacturer instructions. A 320-ng sample of DNA combined with 4 μl of 10 μM primers were sent to EuroFins (Louisville, KY) for Sanger sequencing. Sequences were aligned to the publicly available race and genotype references used by Cianchetta *et al.* (2015). Alignments were performed with Geneious R11 using a global alignment with free end gaps and a 70% BLOSUM cost matrix (Kearse *et al.* 2012). *EF-1α* sequence was insufficient to distinguish FOV race 4 and MDS-12 isolates (Bennett *et al.* 2013); to differentiate between race 4 and MDS-12, the intergenic spacer region was sequenced following the protocol described by Cianchetta *et al.* (2015). Additionally, *EF-1α* sequence cannot distinguish races 1 and 6 (Skovgaard *et al.* 2001). However, reports of race 6 are limited to South America so isolates with an *EF-1α* sequence indicative of race 1 or 6 were assumed to be race 1 (Amstrong and Armstrong 1978; Cianchetta *et al.* 2015).To maximize genetic, temporal, and geographic diversity, 54 *Fusarium oxysporum* isolates were obtained from collections of J. Coleman (Auburn University), R.M. Davis (University of California Davis), and J. Liu (USDA-ARS Southern Plains Agricultural Research Center), as well as the USDA-ARS Northern Regional Research Laboratory (NRRL) culture collection. Forty-eight of the isolates were FOV and 6 were other *formae speciales* of *Fusarium oxysporum* - *Fusarium oxysporum* f. sp. *lycopersici* (*Fol*, NRRL36464; ‘NRRL3′), *Fusarium oxysporum* f. sp. *radicis-lycopersici* (NRRL26379 and NRRL26570; ‘NRRL1’ and ‘NRRL2’, respectively), *Fusarium oxysporum* f. sp. *cubense* (NRRL25609; ‘NRRL4’, and *Fusarium oxysporum* f. sp. *dianthi* (NRRL26960 and NRRL26961; ‘NRRL5′ and ‘NRRL6’, respectively) – all of which are closely related to FOV (O’Donnell *et al.* 2009).

### Genomic DNA extraction and genotyping-by-sequencing (GBS)

Genomic DNA was extracted from a total of 168 single-spore *Fusarium oxysporum* isolates. Isolates were grown on potato dextrose agar (PDA) overlain with sterile cellophane for 6-7 days, after which time mycelia were harvested and lyophilized (Milgroom *et al.* 2014). Approximately 50 milligrams of lyophilized mycelia were frozen in liquid nitrogen and macerated in 2-ml tubes with glass beads in a Geno/Grinder (SPEX SamplePrep, Metuchen, NJ). DNA was extracted using a DNeasy Plant Mini kit (QIAGEN, Valencia, CA) according to manufacturer protocols with the following modification: in the final step of the protocol, samples were eluted in 25 μl AE buffer to increase the final concentration of DNA. Concentration and quality of the DNA were determined using a Nanodrop spectrophotometer ND-1000 (Nanodrop Technologies, Wilmington, DE) and by 0.7% agarose gel electrophoresis, respectively.

Genotyping-by-sequencing was performed following the protocol described by Elshire *et al.* (2011) at the Georgia Genomics and Bioinformatics Core (Athens, GA). Briefly, samples of ≥100 ng genomic DNA were digested with the restriction endonuclease *Ape*KI, ligated with combinatorial barcode adapters, pooled, PCR-amplified, and purified. Samples were then sequenced on an Illumina NextSeq PE150 high output flowcell using 150-bp paired end reads (Illumina, San Diego, CA).

### SNP calling and data filtering

Single nucleotide polymorphisms (SNPs) were identified and called using two modified versions of the uGbS-Flex pipeline (Qi *et al.* 2018). In the first pipeline, SNP calling was performed using a reference-based alignment; in the second, a *de novo* reference was generated from GBS reads following the protocol described by Qi *et al.* (2018) and SNP calling was based on alignment to the *de novo* reference. The rationale for using two separate data analysis pipelines was that the reference genome used in data processing and SNP calling could impact the results of population genetic analyses.

For reference-based SNP calling, quality control was performed using FastQC and raw sequence data were sorted using the process_radtags command in Stacks version 2.0 (Andrews 2010; Catchen *et al.* 2013). Processed reads were trimmed to 120 base pairs and aligned to an annotated reference genome of FOV race 4/7 (NRRL 25433, Broad Institute) using FASTX Trimmer and bowtie2, respectively (Hannon 2010; Langmead and Salzberg 2012). Aligned reads were processed, validated, and sorted using SAMtools and Picard (Broad Institute; Li *et al.* 2009). SNPs were called using GATK HaplotypeCaller, with a ploidy of 1 specified, and GenotypeGVCFs (Van der Auwera *et al.* 2013). SNPs were filtered to retain only biallelic sites, and the resulting VCF file was further filtered for read depth of at least 25, a minimum Phred quality score of 20, and a maximum of 20% missing information per site (Danecek *et al.* 2011).

The *de novo* GBS reference was assembled following the protocol described by Qi *et al.* (2018). Briefly, reads were made equal lengths using FASTX Trimmer, overlapping forward and reverse reads were merged in FLASH, non-overlapping forward and reverse reads were artificially joined and made equal lengths using the in-house python script EL.1.4.py, reads within each sample were clustered using the ustacks function in Stacks version 2.0, and a consensus set of tags from the population was generated with cstacks (Hannon 2010; Catchen *et al.* 2011; Magoc and Salzberg 2011; Qi *et al.* 2018). GBS reads were processed, aligned to the *de novo* reference, and filtered as described above.

### Population genetic analyses

Filtered VCF files were analyzed in R version 3.5.1 with the packages vcfR, poppr 2.0, and adegenet (Jombart and Ahmed 2011; Kamvar *et al.* 2015; Knaus and Grünwald 2017; R Core Team 2018;). A genotype accumulation curve was generated to demonstrate the number of unique SNP genotypes in the population, as well as the minimum number of SNP loci needed to distinguish unique genotypes (Grünwald *et al.* 2003; Kamvar *et al.* 2015). Bitwise genetic distance was calculated and used to construct a minimum spanning network (MSN) with the poppr.msn function, to show the relationships among all individuals in the population (Kamvar *et al.* 2015). Isolates in the minimum spanning network were color-coded according to their race (as inferred from *EF-1α* genotype, and IGS genotype for race 4 and MDS-12) to observe potential clustering patterns by race. Principal component analysis (PCA) was conducted to determine whether isolates of particular races were distinct. PCA was run using the glPca command in adegenet, and results were plotted using the R package ggplot2 (Jolliffe 2002; Jombart and Ahmed 2011; Wickham 2016). Discriminant analysis of principal components (DAPC), or K-means hierarchical clustering, was conducted using the find.clusters command in adegenet in order to determine the optimal number of genetically differentiated clusters across all FOV isolates (Jombart *et al.* 2010).

Additionally, maximum likelihood trees were constructed in MEGA7, using the HKY substitution model with 500 bootstrap replicates (Hasegawa *et al.* 1985; Kumar *et al.* 2016). All analyses were performed on the VCF files generated from the reference-based alignment, and replicated using the VCF file generated from the *de novo*-based alignment to test for differences in results obtained using the two processing methods.

### Data availability

Strains are available upon request. All raw sequence data has been deposited in the NCBI Short Read Archive with project accession number PRJNA632933 and the associated short read accession numbers SRR11792269 – SRR11792418. The supplemental Table S1 lists the isolates included in the population genetic analyses. The supplemental figures show the results of the population genetic analyses using the *de novo*-based SNP dataset: Figure S1 contains the genotype accumulation curve generated from this dataset, Figure S2 contains the maximum likelihood trees, Figure S3 contains the minimum spanning network (MSN) and principal components analysis (PCA), and Figure S4 contains the groups identified through discriminant analysis of principal components (DAPC). Supplemental material available at figshare: https://doi.org/10.25387/g3.12660074.

## Results

### SNP calling, data filtering, and genetic diversity

**Figure 1:**
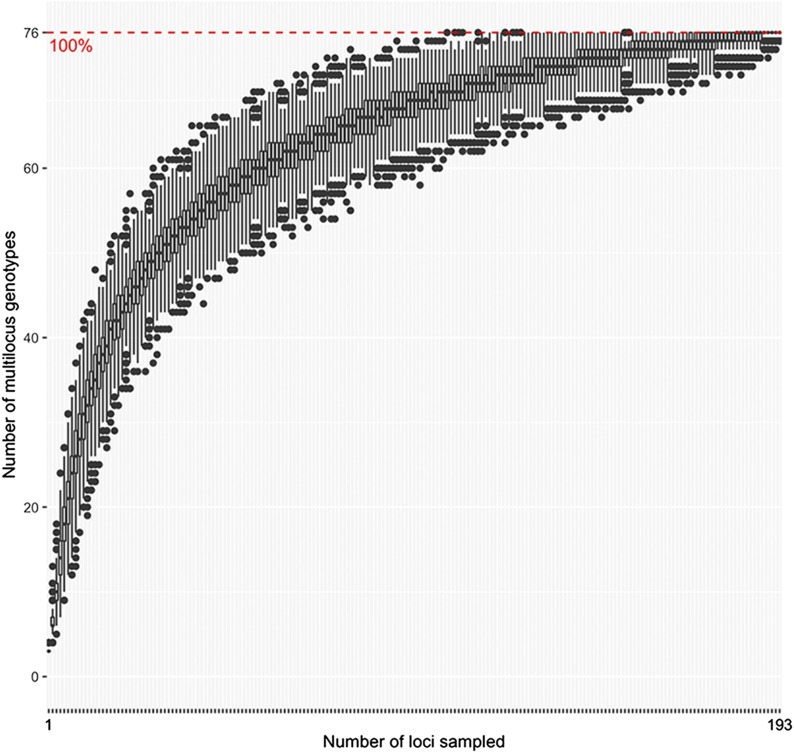
Genotype accumulation curve constructed in poppr (version 2.0), showing the number of unique multilocus genotypes (MLGs) identified in the population of FOV, and the minimum number of SNPs needed to distinguish each MLG.

The unfiltered VCF file based on alignment to the annotated reference genome of NRRL 25433 (Broad Institute) contained 229,338 SNPs. After filtering for read depth, quality, and missing information 193 SNPs remained, and 84 FOV (Table S1) and 6 other *Fusarium oxysporum* isolates were retained for population genetic analyses. Across the 84 FOV isolates that were retained, 76 multilocus genotypes (MLGs) were identified (Figure 1). The genotype accumulation curve generated in poppr never fully plateaued, but the upper end of the 95% confidence interval reached the 100% genotype accumulation curve at approximately 114 SNP loci (Figure 1). The unfiltered VCF file based on alignment to the *de novo* reference contained 1,273 SNPs. The GBS *de novo* reference is very useful for identifying SNP variants, but it is so fragmented that it is not informative in and of itself like the reference genome of NRRL 25433. Since it was generated with GBS reads that are associated with sets of restriction enzymes, the *de novo* assembly consists of 742,892 contigs with A’s added to the end of all reads to make them the same length of approximately 220 bp. After filtering, 374 SNPs, 80 FOV isolates, and 6 other *Fusarium oxysporum* isolates were retained for analyses. All 80 FOV isolates were considered unique MLGs, and the genotype accumulation curve associated with this dataset plateaued at approximately 210 loci (Figure S1).

### Phylogenetic analyses and evolutionary relationships

**Figure 2:**
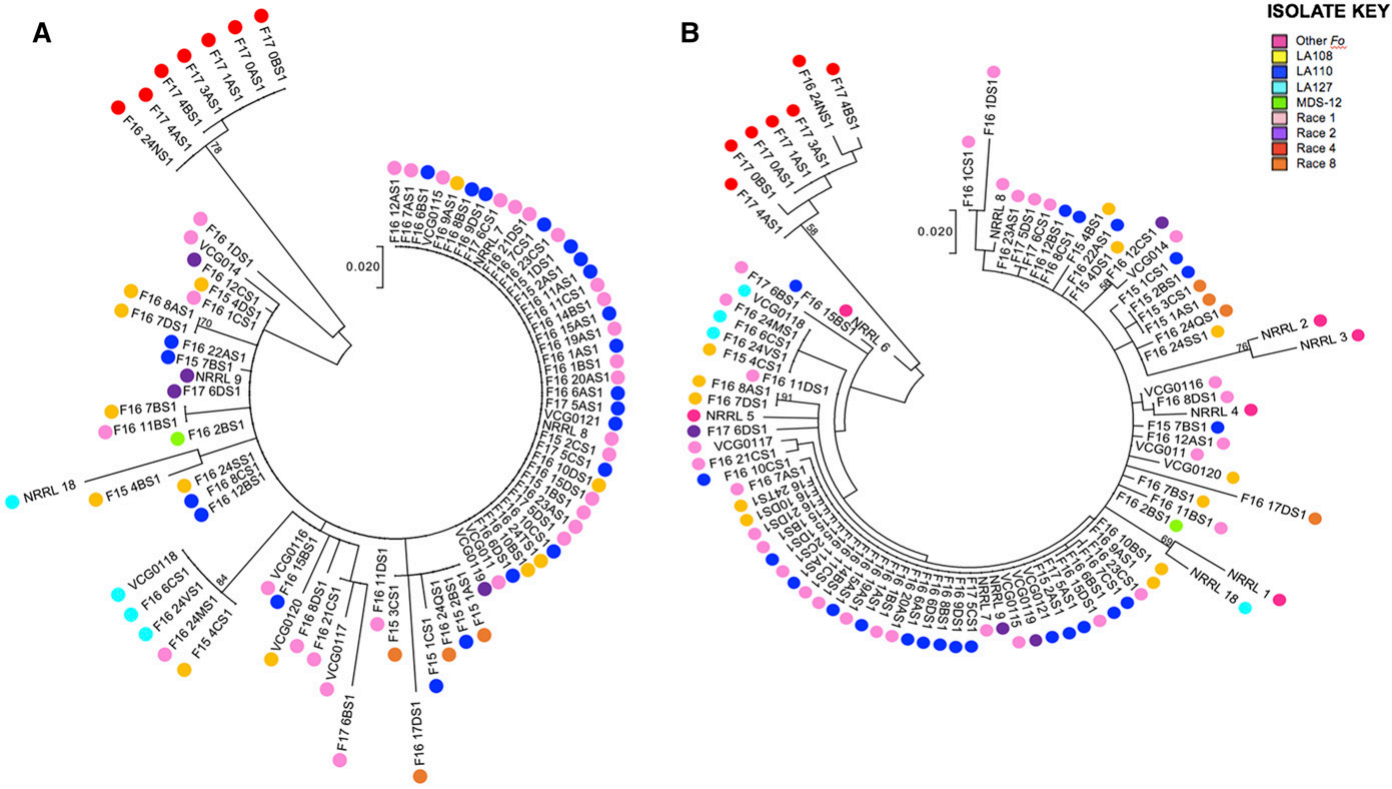
Maximum likelihood (ML) trees constructed in MEGA7 showing the phylogenetic relationships among *Fusarium oxysporum* isolates. The colored circles represent isolates’ *EF-1α*-based races and genotypes, and the numbers on branches represent bootstrap values. (A) Only FOV isolates are included in the ML tree. (B) Six additional (non-FOV) *Fo* isolates are included.

In the ML trees generated from the reference-based SNPs, race 4 isolates were the only FOV to form a monophyletic group according to race or genotype (Figure 2A). The remaining isolates did not cluster by race or *EF-1α* genotype, although most LA127 isolates were placed in the same clade, as were most race 8 isolates. When other *formae speciales* of *Fo* were included in the tree, they did not form an outgroup or monophyletic clade, although two of these *Fo* isolates (‘NRRL2’ and ‘NRRL3′, which correspond to *F. oxysporum* f. sp. *radicis-lycopersici* NRRL26570 and *F. oxysporum* f. sp. *lycopersici* NRRL36464, respectively) did cluster together on a branch with bootstrap support of 76 (Figure 2B). The ML tree based on alignment to the *de novo* reference was similar but not identical to the tree described above: FOV race 4 isolates still clustered together on a branch with high bootstrap support, but there were no phylogenetically informative differences displayed among the race 4 isolates (Figure S2A; Figure S2B). Additionally, more clustering by race was observed in the *de novo*-based trees, regardless of whether other *Fo* isolates were included in the analysis. In addition to the LA127 isolates and race 8 isolates generally grouping together as before, four LA108 isolates formed a monophyletic group, and all four race 2 isolates were placed in the same clade; however, the bootstrap support for these new groups was below 50 (Figure S2A; Figure S2B).

### Population structure

**Figure 3:**
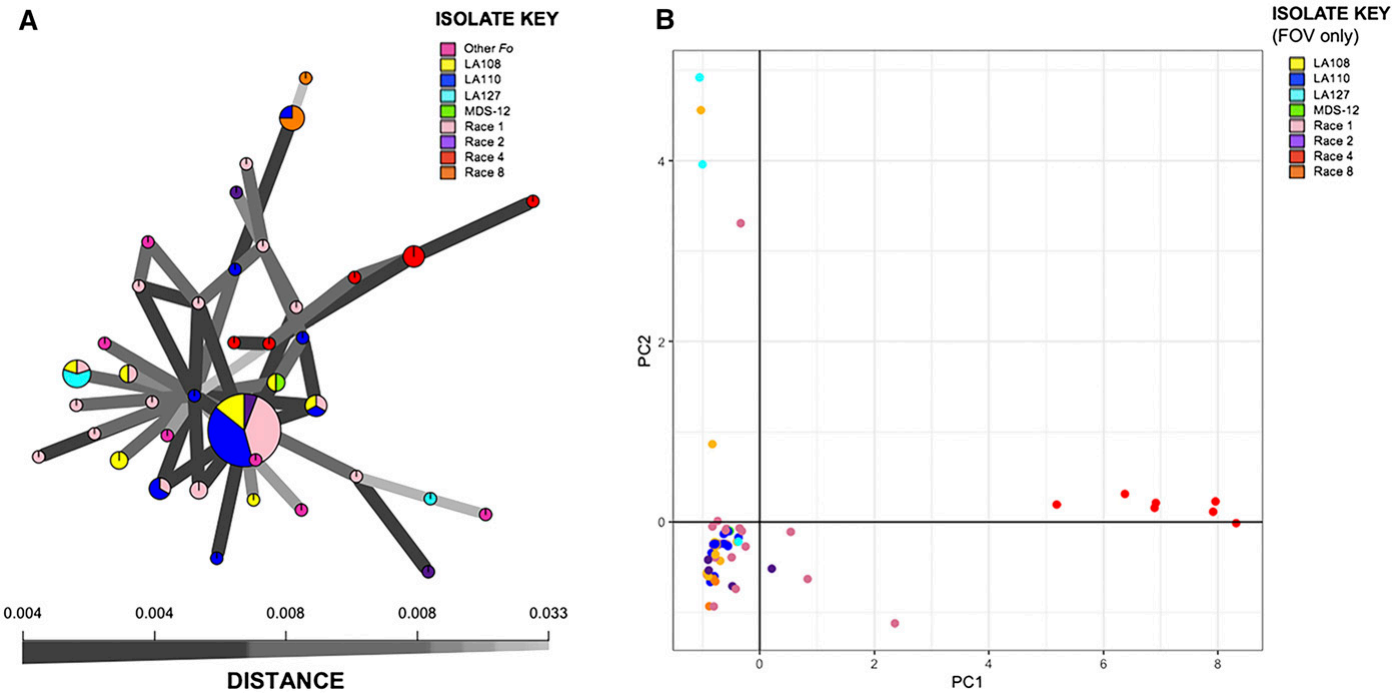
Population genetic structure. (A) Minimum-spanning network (MSN) constructed in poppr (version 2.0). Six additional (non-FOV) *Fo* isolates are included in this figure. (B) Principal component analysis (PCA) was conducted using poppr (version 2.0).

In the minimum spanning network (MSN) constructed from the reference-based SNP dataset, isolates did not cluster neatly by race or genotype (Figure 3A). Other *formae speciales* of *Fo* were included in the MSN, and while all non-FOV isolates were distantly related from most individuals in the population, they did not cluster together (Figure 3A). The PCA analysis demonstrated a similar pattern as the MSN but showed a clear grouping of race 4 isolates, which were separated from most other isolates in the population by PC1 and PC2 (Figure 3B). Most isolates in the population overlapped each other in the lower left-hand quadrant.

The MSN constructed from the *de novo*-based SNP dataset showed more clustering by race than the reference-based dataset, with most race 2 isolates and LA127 isolates clustering together (Figure S3A). Additionally, all race 4 isolates formed a single, undifferentiated cluster in the *de novo*-based MSN (Figure S3A). The *de novo*-based PCA was highly similar to the reference-based analysis, but race 4 isolates were more dispersed and overlapped with a race 1 isolate (Figure S3B).

**Figure 4:**
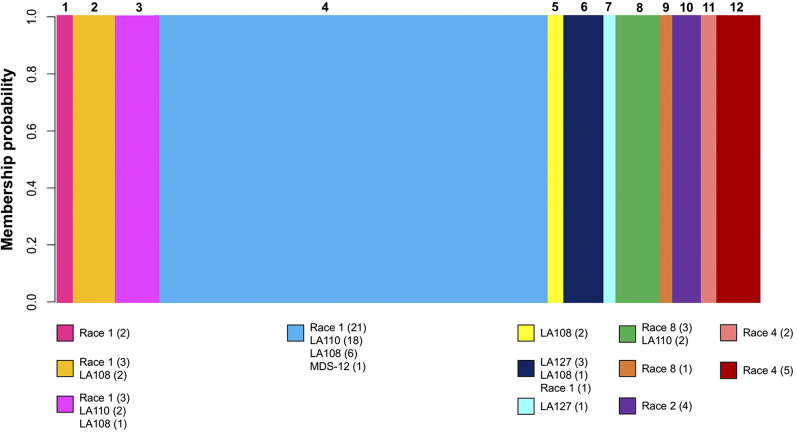
Twelve genetically distinct groups of FOV identified through K-means hierarchical clustering.

Using the reference-based SNPs, DAPC identified an optimum of k = 12 genetically distinct clusters (Figure 4). All individuals had a 100% membership probability to the group which they were assigned. All clusters contained between one and six individuals, except for group 4 which contained 46 individuals. There were 20 SNPs within the first 6 PC that played the largest role in DAPC group determination. Some clusters contained isolates of a single race or *EF-1α* genotype; for example, group 1 contained only race 1 isolates, group 10 contained only race 2 isolates, and groups 11 and 12 contained only race 4 isolates. However, some races spanned multiple groups and, conversely, some groups contained individuals of multiple races and genotypes. For example, race 1 and LA108 isolates were both present in five of the twelve groups identified; and group 4 included isolates spanning race 1, LA110, LA108, and MDS-12.

When DAPC was run on the *de novo*-based SNPs, an optimum of k = 9 clusters were identified (Figure S4). As with the reference-based analysis, all individuals were assigned to groups with 100% membership probability. Some groups identified in this analysis were identical to those identified in the reference-based DAPC analysis. For example, group 1 was the same across both analyses, and group 5 in the reference-based analysis was identical to group 7 in the reference-based analysis. Some groups identified in the *de novo*-based analysis were analogous, although not quite identical, to groups identified in the reference-based analysis: an example of this is group 4, which contained 51 individuals spanning five races and genotypes in the *de novo*-based analysis.

## Discussion

Using a genotyping-by-sequencing (GBS) approach, we identified novel genetic diversity in the cotton wilt pathogen *Fusarium oxysporum* f. sp. *vasinfectum* (FOV). In our reference-based data processing pipeline, we identified 193 SNPs and 76 unique multilocus genotypes (MLGs) of FOV, and found that greater than 193 SNPs are most likely needed to fully distinguish unique genotypes of FOV in the population. However, the upper end of the 95% confidence interval did not plateau in this analysis, demonstrating that 193 SNPs are insufficient to distinguish the full genetic diversity of FOV, although 114 SNPs are sufficient to capture at least 95% of the population’s diversity. In the *de novo*-based analysis, we identified 374 SNPs and 80 MLGs, and found that 210 SNP loci were needed to distinguish unique genotypes. The results of the reference-based and *de novo*-based analyses complement each other, and suggest that 210 is a realistic minimum number of SNPs needed to conduct high-resolution population genetic analyses of FOV. However, some of the SNPs identified in the *de novo*-based data analysis pipeline may be redundant, as the UGbS-Flex pipeline neither assessed redundancy nor removed redundant SNPs (Qi *et al.* 2018). Additionally, MLGs are not straightforward to interpret in the context of datasets with hundreds to thousands of SNPs - some of the unique MLGs identified in our study were different only by one or a few SNPs, which may not warrant classification as different genotypes (Grünwald *et al.* 2016). The concept that MLGs are problematic for SNP genotypes is further demonstrated by the fact that all 80 individuals were considered unique MLGs in the *de novo*-based genotype accumulation curve, which was based on a larger number of SNPs than the reference-based genotype accumulation curve. Although the exact number of MLGs in the population is unclear, our genotype accumulation curves suggest that using one or a few housekeeping genes does not provide enough resolution for population analyses of FOV, and that such an approach is likely to overlook genetic diversity. Our DAPC results provide additional evidence that FOV is more genetically diverse than its races and *EF-1α* genotypes: only eight races and *EF-1α* genotypes were represented among the isolates analyzed by DAPC, but an optimum of k = 12 and k = 9 genetically distinct groups of FOV were identified using reference-based SNPs and *de novo*-based SNPs, respectively. Although our population genetic analyses provide new evidence that FOV is more diverse than its races and *EF-1α* genotypes, we are not the first to suggest this. The U.S. cotton industry uses races and genotypes based on *EF-1α* sequence to characterize FOV isolates, but FOV can also be classified into vegetative compatibility groups (VCGs) (Bell *et al.* 2017). Vegetative compatibility is defined by the ability of isolates to undergo anastomosis, or hyphal fusion, and isolates are able to undergo anastomosis if they have identical alleles at several *vic* loci (Leslie 1993). Because individuals in the same VCG must possess several identical alleles, vegetative compatibility is thought to be a good indication of isolates’ relatedness (Puhalla 1985). Additionally, Bell *et al.* (2017) reported that individuals in the same VCG typically possess the same disease phenotype on cotton, suggesting that VCG is an ideal way to characterize FOV isolates as it is indicative of both genetic relationship and disease phenotype. Twenty distinct VCG’s have been identified in FOV (Fernandez *et al.* 1994; Davis *et al.* 1996; Bell *et al.* 2017), but our study identified a maximum of twelve genetically distinct groups of FOV. One possible explanation for this is that some VCG’s are rare - for example, between 1994 and 2017 only one isolate belonging to the VCG known as 0111 had been identified (Fernandez *et al.* 1994; Bell *et al.* 2017) - and therefore were not represented among the isolates analyzed in our study. Additionally, it is possible that some FOV isolates differ at *vic* alleles which govern vegetative compatibility but are otherwise genetically similar, resulting in individuals of different VCGs being placed in the same DAPC group in our study. Although VCG typing is quite useful for identifying and characterizing genetic diversity within FOV, it is labor-intensive and not conducive to rapid characterization of genetic diversity in larger populations.

Our results also demonstrated that FOV race 4 isolates comprise a genetically distinct group, whereas the relationships among all other FOV isolates are not explained by their race or *EF-1α* genotype. This was demonstrated in maximum likelihood trees in which race 4 isolates formed a monophyletic group on a longer branch with high bootstrap support in both analyses, while the other races and genotypes of FOV were not monophyletic and their depicted relationships generally had bootstrap support below 50. The lack of race or *EF-1α* genotype explaining overall genetic relatedness among populations of FOV was also demonstrated in the minimum spanning network (MSN) and principal components analysis (PCA), which showed a clear clustering of race 4 isolates, but little or incomplete clustering of other races or genotypes. Additionally, the clusters identified by DAPC were also not structured by race or *EF-1α* genotype. Some clusters contained individuals of multiple races or genotypes, such as group 4 in the reference-based analysis, which contained 46 individuals spanning race 1, LA108, LA110, and MDS-12; this suggests that isolates with different *EF-1α* genotypes can possess a high degree of genetic similarity. Conversely, some races or genotypes based on *EF-1α* sequence were present in multiple genetic clusters: in the reference-based analysis, race 1 isolates were present in five of twelve clusters, showing that individuals sharing the same *EF-1α* sequence are not necessarily closely related. Taken together, our population analyses provide compelling evidence that race or *EF-1α* genotype is not reflective of FOV isolates’ genetic relatedness, with the exception of race 4.

The finding that race or *EF-1α* sequence is generally not indicative of isolates’ genetic relatedness leads to the question of whether *EF-1α* genotypes are biologically meaningful. In previous studies, certain *EF-1α* genotypes have been associated with disease phenotypes. FOV race 4, for example, is considered the most virulent race and is associated with early-season damage and vascular discoloration of the taproot (Kim *et al.* 2005; Cianchetta *et al.* 2015). Races 1 and 2 have been described as generally mild, and characterizations of MDS-12 isolates range from non-virulent to highly aggressive (Cianchetta *et al.* 2015; Bell *et al.* 2017). Since *EF-1α* genotypes do not reflect the full genetic diversity or population structure of FOV, their reported disease phenotypes should be further investigated to determine if there is any biological meaning associated with *EF-1α* genotypes. The SNPs identified in this study should also be evaluated for association with disease phenotype, to determine if FOV would be better characterized by SNP genotype than *EF-1α*-based race or genotype.

Finally, this study is the first that we know of to assess the effect of the reference genome used in sequence alignment on the results of population analyses. Using the same SNP-calling and filtering parameters in both our reference-based and *de novo*-based data processing pipelines, more SNPs were retained for population genetic analyses using the *de novo*-based approach: 374 SNPs were retained using this approach, as compared to only 193 SNPs with the reference-based approach. This may suggest that using a consensus reference leads to more robust population analyses, especially for a high-diversity species like FOV, although it is currently unclear if any of the SNPs identified in our *de novo*-based reference are redundant. However, a major limitation of the *de novo* assembled reference genome was that the unfiltered SNP dataset based on this reference only contained 1,273 SNPs, whereas the unfiltered SNP dataset based on the race 4 reference genome contained 229,338 SNPs. It is unclear why the unfiltered *de novo*-based SNP dataset contained so few SNPs; this could be a result of the parameters that were used to construct the *de novo* reference. While we used the UGbs-Flex protocol to assemble a consensus reference *de novo* from the raw GBS reads, a consensus reference can also be constructed by mapping consensus reads to a reference genome (Li *et al.* 2009); this approach might result in more SNPs being yielded in the unfiltered SNP dataset. Another important distinction between the two data-analysis pipelines was that the reference-based population genetic analyses, especially the minimum spanning network, showed increased differentiation of FOV race 4 isolates, which comprised only 8% of isolates analyzed, but less differentiation among the most prevalent races and genotypes in the population (race 1, LA108, and LA110); while the *de novo*-based population genetic analyses showed more differentiation among common races and less differentiation among race 4 isolates. The differences we observed between the two pipelines may be the result of the reference genome being a race 4/7 isolate, which we have found to be genetically distinct from other FOV races using both methods. Importantly, both the reference-based and *de novo*-based analyses showed similar results; their main distinction was the amount of differentiation observed among race 1, race 4, LA108, and LA110 isolates. These slight but important differences could mean that in population genomic analyses of high-diversity species, results are skewed toward increased differentiation of isolates that are genetically similar to the reference genome used for alignment and SNP calling.

## Conclusions

In summary, we demonstrated through population genetic analyses that FOV is not structured by *EF-1α* genotype, which is currently used to assign isolates to race and make assumptions about their virulence. We also found new evidence supporting the hypothesis that FOV is more genetically diverse than what is reported based on race or *EF-1α* genotype. Furthermore, we found that the reference genome used in sequence alignment and SNP calling influences the results of population genomic analyses: for a high-diversity organism like FOV, using a consensus reference seems to yield more high-quality SNPs with which to conduct population analyses, and results in increased differentiation of the most prevalent genotypes in the population. It is currently unclear how the genetic diversity and population structure of FOV relate to the pathogen’s disease phenotypes; this should be investigated in order to characterize FOV in a biologically meaningful way and improve Fusarium wilt management.

## References

[bib1] Anderson J. P., Gleason C. A., Foley R. C., Thrall P. H., Burdon J. B., 2010 Plants *vs.* pathogens: an evolutionary arms race. Func. Plant Biol. 37: 499–512. 10.1071/FP09304 PMC3131095 21743794

[bib2] Andrews K. R., Good J. M., Miller M. R., Luikart G., and Hohenlohe P. A., 2016 Harnessing the power of RADseq for ecological and evolutionary genomics. Nat. Rev. Genet. 17: 81–92. 10.1038/nrg.2015.28 26729255 PMC4823021

[bib3] Andrews S., 2010 FastQC a quality control tool for high throughput sequence data. http://www.bioinformatics.babraham.ac.uk/projects/fastqc/

[bib4] Armstrong G. M., and Armstrong J. K., 1958 A race of the cotton-wilt *Fusarium* causing wilt of Yelredo soybean and flue-cured tobacco. Plant Dis. Rep. 42: 1147–1151.

[bib5] Armstrong, G. M., and J. K. Armstrong, 1960 American, Egyptian, and Indian cotton wilt Fusaria: their pathogenicity and relationship to other wilt Fusaria. U. S. Dep. Agric. Tech. Bull. 1219: 1–19.

[bib6] Armstrong G. M., and Armstrong J. K., 1978 A new race (race 6) of the cotton-wilt *Fusarium* from Brazil. Plant Dis. Rep. 62: 421–423.

[bib7] Atkinson G. F., 1892 Some diseases of cotton. Bull. Ala. Agric. Exp. Station 41: 19–29.

[bib8] Bell A. A., Kemerait R. C., Ortiz C. S., Prom S., Quintana J., 2017 Genetic diversity, virulence, and *Meloidogyne incognita* interactions of *Fusarium oxysporum* isolates causing cotton wilt in Georgia. Plant Dis. 101: 948–956. 10.1094/PDIS-09-16-1382-RE 30682930

[bib9] Bennett R. S., Scott T. Z., Lawrence K. S., and Lawrence G. W., 2013 Sequence characterization of race 4-like isolates of *Fusarium oxysporum* from Alabama and Mississippi. J. Cotton Sci. 17: 125–130.

[bib10] Broad Institute, 2018 Picard Tools. http://broadinstitute.github.io/picard/

[bib11] Catchen J., Amores A., Hohenlohe P., Crezko W., and Postlethwait J. H., 2011 Stacks: building and genotyping loci *de novo* from short-read sequences. G3 (Bethesda) 1: 171–182. 10.1534/g3.111.000240 22384329 PMC3276136

[bib12] Catchen J., Hohenlohe P. A., Bassham S., Amores A., and Cresko W. A., 2013 Stacks: an analysis tool set for population genomics. Mol. Ecol. 22: 3124–3140. 10.1111/mec.12354 23701397 PMC3936987

[bib13] Chen Q., Ji X., and Sun W., 1985 Identification of races of cotton wilt *Fusarium* in China. Agric. Sci. China 6: 1–6.

[bib14] Cianchetta A. N., Allen T. W., Hutmacher R. B., Kemerait R. C., Kirkpatrick T. L., 2015 Survey of *Fusarium oxysporum* f. sp. *vasinfectum* in the United States. J. Cotton Sci. 19: 328–336.

[bib15] da Silva M. B., Davis R. F., Doan H., Nichols R. L., Kemerait R. C., 2019 Fusarium wilt of cotton may commonly result from the interaction of *Fusarium oxysporum* f. sp. *vasinfectum* with *Belonolaimus longicaudatus*. J. Nematol. 51: 1–10. 10.21307/jofnem-2019-015 PMC6929639 31088027

[bib16] de Sain M., and Rep M., 2015 The role of pathogen-secreted proteins in fungal vascular wilt diseases. Int. J. Mol. Sci. 16: 23970–23993. 10.3390/ijms161023970 26473835 PMC4632733

[bib17] Danecek P., Auton A., Abecasis G., Albers C. A., Banks E., 2011 The variant call format and VCFtools. Bioinformatics 27: 2156–2158. 10.1093/bioinformatics/btr330 21653522 PMC3137218

[bib18] Davis R. D., Moore N. Y., and Kochman J. K., 1996 Characterisation of a population of *Fusarium oxysporum* f. sp. *vasinfectum* causing wilt of cotton in Australia. Aust. J. Agric. Res. 47: 1143–1156. 10.1071/AR9961143

[bib19] Davis R. M., Colyer P. D., Rothrock C. S., and Kochman J. K., 2006 Fusarium wilt of cotton: population diversity and implications for management. Plant Dis. 90: 692–703. 10.1094/PD-90-0692 30781226

[bib21] Edel-Hermann V., and Lecomte C., 2019 Current status of *Fusarium oxysporum formae speciales* and races. Phytopathology 109: 512–530. 10.1094/PHYTO-08-18-0320-RVW 30461350

[bib66] Elshire R. J., Glaubitz J. C., Sun Q., Poland J. A., Kawamoto K., 2011 A robust, simple genotyping-by-sequencing (GBS) approach for high diversity species. PLoS ONE. 6: e19379 10.1371/journal.pone.0019379 21573248 PMC3087801

[bib23] Fernandez D., Assigbetse K., Dubois M. P., and Geiger J. P., 1994 Molecular characterization of races and vegetative compatibility groups in *Fusarium oxysporum* f. sp. *vasinfectum*. Appl. Environ. Microbiol. 60: 4039–4046. 10.1128/AEM.60.11.4039-4046.1994 7993090 PMC201933

[bib24] Garber R. H., and Paxman G. A., 1963 Fusarium wilt of cotton in California. Plant Dis. Rep. 47: 398–400.

[bib65] Garber R. H., Jorgenson E. C., Smith S., and Hyer A. H., 1979 Interaction of population levels of *Fusarium oxysporum* f. sp. *vasinfectum* and *Meloidogyne incognita* on cotton. J. Nemat. 11: 133–137. PMC2617958 19305546

[bib26] Grünwald N. J., Goodwin S. B., Milgroom M. G., and Fry W. E., 2003 Analysis of genotypic diversity data for populations of microorganisms. Phytopathology 93: 738–746. 10.1094/PHYTO.2003.93.6.738 18943061

[bib27] Grünwald N. J., McDonald B. A., and Milgroom M. G., 2016 Population genomics of fungal and oomycete pathogens. Annu. Rev. Phytopathol. 54: 323–346. 10.1146/annurev-phyto-080614-115913 27296138

[bib28] Hannon, G. J., 2010 FASTX-Toolkit. http://hannonlab.cshl.edu/fastx_toolkit

[bib29] Hansen Z. R., Everts K. L., Fry W. E., Gevens A. J., Grünwald N. J., 2016 Genetic variation within clonal lineages of *Phytophthora infestans* revealed through genotyping-by-sequencing, and implications for late blight epidemiology. PLoS One 11: e0165690 10.1371/journal.pone.0165690 27812174 PMC5094694

[bib30] Hasegawa M., Kishino H., and Yano T., 1985 Dating of the human-ape splitting by a molecular clock of mitochondrial DNA. J. Mol. Evol. 22: 160–174. 10.1007/BF02101694 3934395

[bib31] Hering O., Nirenberg H. I., Kohn S., and Deml G., 1999 Characterization of isolates of *Fusarium oxysporum* f. sp. *vasinfectum* (Atk.) Snyd. & Hans., races 1–6, by cellular fatty acid analysis. J. Phytopathol. 147: 509–514. 10.1111/j.1439-0434.1999.tb03857.x

[bib32] Holmes E. A., Bennett R. S., Spurgeon D. W., Colyer P. D., and Davis R. M., 2009 New genotypes of *Fusarium oxysporum* f. sp. *vasinfectum* from the Southeastern United States. Plant Dis. 93: 1298–1304. 10.1094/PDIS-93-12-1298 30759505

[bib33] Ibrahim G., 1966 A new race of the cotton-wilt *Fusarium* in the Sudan Gezira. Emp. Cotton Grow. Rev. 43: 296–299.

[bib34] Jolliffe I. T., 2002 Principal Component Analysis, Ed. 2nd Springer, New York.

[bib35] Jombart T., Devillard S., and Balloux F., 2010 Discriminant analysis of principal components: a new method for the analysis of genetically structured populations. BMC Genet. 11: 94 10.1186/1471-2156-11-94 20950446 PMC2973851

[bib36] Jombart T., and Ahmed I., 2011 *adegenet 1.3–1*: new tools for the analysis of genome-wide SNP data. Bioinformatics 24: 1403–1405. 10.1093/bioinformatics/btn129 PMC3198581 21926124

[bib64] Jorgenson E. C., Hyer A. H., Garber R. H., and Smith S. N., 1978 The influence of soil fumigation on the Fusarium root-knot-nematode complex of cotton in California. J. Nematol. 10: 228–231. 19305846 PMC2617888

[bib37] Kamvar Z. N., Brooks J. C., and Grünwald N. J., 2015 Novel R tools of genome-wide population genetic data with emphasis on clonality. Front. Genet. 6: 208 10.3389/fgene.2015.00208 26113860 PMC4462096

[bib63] Kang S., Demers J., Jimenez-Gasco M., and Rep M., 2014 *Fusarium oxysporum *p. 99–119 *In:* R. A. Dean *et al.* (eds). *Genomics of Plant-Associated Fungi and Oomycetes: Dicot Pathogens*. Springer, New York.

[bib38] Kearse M., Moir R., Wilson A., Stones-Havas S., Cheung M., 2012 Geneious Basic: an integrated and extendable desktop software platform for the organization and analysis of sequence data. Bioinformatics 28: 1647–1649. 10.1093/bioinformatics/bts199 22543367 PMC3371832

[bib39] Kim Y., Hutmacher R. B., and Davis R. M., 2005 Characterization of California isolates of *Fusarium oxysporum* f. sp. *vasinfectum*. Plant Dis. 89: 366–372. 10.1094/PD-89-0366 30795451

[bib40] Knaus B. J., and Grünwald N. J., 2017 *VcfR*: Apackage to manipulate and visualize Variant Call format data in R. Mol. Ecol. Resour. 17: 44–53. 10.1111/1755-0998.12549 27401132

[bib43] Kumar S., Stecher G., and Tamura K., 2016 MEGA7: Molecular Evolutionary Genetics Analysis version 7.0 for bigger datasets. Mol. Biol. Evol. 33: 1870–1874. 10.1093/molbev/msw054 27004904 PMC8210823

[bib44] Langmead B., and Salzberg S. L., 2012 Fast gapped read alignment with Bowtie 2. Nat. Methods 9: 357–359. 10.1038/nmeth.1923 22388286 PMC3322381

[bib62] Leslie J. F., 1993 Fungal vegetative compatibility. Annu. Rev. Phytopathol. 31: 127–150. 18643765 10.1146/annurev.py.31.090193.001015

[bib45] Li H., Handsaker B., Wysoker A., Fennell T., Ruan J., 2009 The Sequence Alignment/Map format and SAMtools. Bioinformatics 25: 2078–2079. 10.1093/bioinformatics/btp352 19505943 PMC2723002

[bib46] Ma L. J., van der Does C. H., Borkovich K. A., Coleman J. J., Daboussi M. J., 2010 Comparative genomics reveals mobile pathogenicity chromosome in *Fusarium*. Nature 464: 367–373. 10.1038/nature08850 20237561 PMC3048781

[bib47] Magoc T., and Salzberg S., 2011 FLASH: fast length adjustment of short reads to improve genome assemblies. Bioinformatics 27: 2957–2963. 10.1093/bioinformatics/btr507 21903629 PMC3198573

[bib48] Martyn R. D., 2014 Fusarium wilt of watermelon: 120 years of research. Horticultural Reviews 42: 349–442. 10.1002/9781118916827

[bib49] Milgroom M. G., Jimenez-Gasco M., Olivares-Garcia C., Drott M. T., and Jimenez-Diaz R. M., 2014 Recombination between clonal lineages of the asexual fungus *Verticillium dahliae* detected by genotyping by sequencing. PLoS One 9: e106740 10.1371/journal.pone.0106740 25181515 PMC4152335

[bib50] Nirenberg H. I., Ibrahim G., and Michail S. H., 1994 Race identify of three isolates of *Fusarium oxysporum* f. sp. *vasinfectum* (Atk.) Snyd. & Hans. from Egypt and the Sudan. Z. Pflanzenkr. Pflanzenschutz 101: 594–597.

[bib51] O’Donnell K., Kistler H. C., Cigelnik E., and Ploetz R. C., 1998 Multiple evolutionary origins of the fungus causing Panama disease of banana: concordant evidence from nuclear and mitochondrial gene genealogies. Proc. Natl. Acad. Sci. USA 95: 2044–2049. 10.1073/pnas.95.5.2044 9482835 PMC19243

[bib52] O’Donnell K., Gueidan C., Sink S., Johnston P. R., Crous P. W., 2009 A two-locus DNA sequence database for typing plant and human pathogens within the *Fusarium oxysporum* species complex. Fungal Genet. Biol. 46: 936–948. 10.1016/j.fgb.2009.08.006 19715767

[bib53] Ploetz R. C., 1994 Panama disease: Return of the first banana menace. Int. J. Pest Manage. 40: 326–336. 10.1080/09670879409371908

[bib61] Puhalla J. E., 1985 Classification of strains of Fusarium oxysporum on the basis of vegetative compatibility. Can. J. Botany 63: 179–183.

[bib54] Qi P., Gimode D., Saha D., Schroder S., Chakraborty D., 2018 UGbs-Flex, a novel bioinformatics pipeline for imputation-free SNP discovery in polyploids without a reference genome: finger millet as a case study. BMC Plant Biol. 18: 117–135. 10.1186/s12870-018-1316-3 29902967 PMC6003085

[bib55] R Core Team, 2018 R: A language and environment for statistical computing. Vienna, Austria. URL https://www.R-project.org/.

[bib56] Skovgaard K., Nirenberg H. I., O’Donnell K., and Rosendahl S., 2001 Evolution of *Fusarium oxysporum* f. sp. *vasinfectum* races inferred from multigene genealogies. Phytopathology 91: 1231–1237. 10.1094/PHYTO.2001.91.12.1231 18943339

[bib57] Talas F., Kalih R., Miedaner T., and McDonald B. A., 2016 Genome-wide association study identifies novel candidate genes for aggressiveness, deoxynivalenol production, and azole sensitivity in a natural field population of *Fusarium graminearum*. Mol. Plant Microbe Interact. 29: 417–430. 10.1094/MPMI-09-15-0218-R 26959837

[bib58] Van der Auwera G. A., Carneiro M. O., Hartl C., Poplin R., Del Angel G., 2013 From FastQ data to high confidence variant calls: the Genome Analysis Toolkit best practices pipeline. Curr. Protoc. Bioinformatics 43: 1–33. 25431634 10.1002/0471250953.bi1110s43 PMC4243306

[bib59] Whitaker, J., S. Culpepper, G. Harris, R. C. Kemerait, C. Perry *et al.*, 2016 Georgia Cotton Production Guide. The University of Georgia Cooperative Extension. www.ugacotton.com

[bib60] Wickham H., 2016 ggplot2: Elegant Graphics for Data Analysis. Springer, New York.

